# Comprehensive Analysis of lncRNA Expression Profile and the Potential Role of ENST00000604491 in Graves' Disease

**DOI:** 10.1155/2022/8067464

**Published:** 2022-04-25

**Authors:** Yingzhao Liu, Junli Zou, Juan Xu, Xuehua Wang, Jie Xing, Li Wang, Huiyong Peng

**Affiliations:** ^1^Department of Endocrinology, The Affiliated People's Hospital of Jiangsu University, Zhenjiang Medical School of Nanjing Medical University, Zhenjiang 212002, China; ^2^Department of Critical Care Medicine, The Affiliated People's Hospital of Jiangsu University, Zhenjiang Medical School of Nanjing Medical University, Zhenjiang 212002, China; ^3^Department of Endocrinology, The Fourth Affiliated Hospital of Jiangsu University, Zhenjiang 212001, China; ^4^Department of Laboratory Medicine, The Affiliated People's Hospital of Jiangsu University, Zhenjiang Medical School of Nanjing Medical University, Zhenjiang 212002, China; ^5^Department of Genetic Toxicology, The Key Laboratory of Modern Toxicology of Ministry of Education, Center for Global Health, School of Public Health, Nanjing Medical University, Nanjing 211100, China

## Abstract

**Background:**

Graves' disease (GD) is one of the most common autoimmune diseases worldwide and develops in 20 to 50 cases per 100,000 persons annually. Long noncoding RNAs (lncRNAs) are widely expressed in multiple human diseases and have pivotal functions in gene regulation. This study is aimed at determining the lncRNA profile in peripheral blood mononuclear cells (PBMCs) from GD patients and investigating the role of ENST00000604491 in GD.

**Methods:**

A total of 31 GD patients and 32 normal controls were enrolled in the study. Next-generation sequencing was performed to identify the dysregulated lncRNAs in the PBMCs from the 5 GD patients and 5 normal controls, and 26 GD patients and 27 controls were used to verify the selected lncRNAs. The relative expression of verified lncRNAs, forkhead box P1 (FOXP1), and IKAROS family zinc finger 3 (IKZF3) from these samples was detected by quantitative real-time PCR. The potential biomarker value was assessed by using receiver operating characteristic (ROC) curve analysis.

**Results:**

A total of 37,683 dysregulated expressed lncRNAs were indicated, of which 5 lncRNAs were significantly upregulated and 83 lncRNAs were remarkably downregulated in the GD patients compared with healthy subjects. Gene Ontology and Kyoto Encyclopedia of Genes and Genomes pathway analyses showed that abnormally expressed lncRNAs were mainly enriched in immune system-related signalling pathways. Among the selected lncRNAs, the relative expression of ENST00000604491 was significantly downregulated and negatively correlated with the serum levels of thyroid-stimulating hormone receptor antibodies (TRAb) in GD patients. Further studies confirmed that decreased FOXP1 expression was inversely correlated with serum TRAb levels in GD patients. Moreover, there was a notably positive correlation between ENST00000604491 expression and FOXP1 transcript levels in GD. The area under the ROC curve of ENST00000604491 was up to 0.74 (95% confidence interval: 0.60-0.87, *p* < 0.01), and the sensitivity and specificity were 53.85% and 88.89%, respectively.

**Conclusion:**

The present study identifies ENST00000604491 as a significantly attenuated lncRNA in GD patients, which may contribute to the pathogenesis of GD by regulating FOXP1 and represent a potential biomarker for GD.

## 1. Introduction

Graves' disease (GD) is a chronic autoimmune thyroid disease associated with multiple body systems [1]. It is the most common cause of hyperthyroidism, accounting for ~80% of all cases of hyperthyroidism and affecting approximately 1% of the general population [2, 3]. People can be affected at any age, and females have a higher morbidity rate [4]. The clinical symptoms of GD are mainly characterized by hypermetabolic syndrome, diffuse goiter, pretibial myxoedema, and ophthalmopathy [5]. Abnormal secretion of thyroid-associated autoantibodies is the most prominent immunological sign of the disease, of which thyroid-stimulating hormone receptor antibodies (TRAb) are more valuable for GD diagnosis [1]. Although genetic and epigenetic alterations are leading candidates for the factors contributing to the aetiology of GD [6], the pathogenesis of this enigmatic thyroid disease remains elusive.

Long noncoding RNAs (lncRNAs) are a group of transcripts greater than 200 nucleotides in length that do not encode functional proteins [7]. The GENCODE database (version 39) annotates 18,811 human lncRNA genes and 53,009 human lncRNA transcripts [8]. These lncRNAs are classified as sense lncRNAs, antisense lncRNAs, bidirectional lncRNAs, lincRNAs, and intronic lncRNAs based on their localization in the genome [9]. lncRNAs are decisive in activating or suppressing gene expression and have been implicated in the initiation and progression of various diseases [10, 11]. Evidence has suggested that many lncRNAs are involved in the pathogenesis of autoimmune diseases, including rheumatoid arthritis, systemic lupus erythematosus, and Hashimoto's thyroiditis [12–14]. Although the lncRNA profiles in CD4^+^ T cells of relapsed GD have been preliminarily investigated [15, 16], the dysregulated lncRNAs in peripheral blood mononuclear cells (PBMCs) from GD patients and their potential functions remain poorly understood. Here, the present study is aimed at investigating the potential roles of lncRNAs in PBMCs from patients with GD.

## 2. Materials and Methods

### 2.1. Subjects and Samples

A total of 26 adult patients with GD, including 20 females and 6 males, and 27 adult age- and sex-matched normal controls (NCs), including 20 females and 7 males, were enrolled from the Affiliated People's Hospital of Jiangsu University. The diagnosis of GD was based on characteristic clinical features and biochemical abnormalities. Among them, 16 GD patients were newly diagnosed and 10 GD patients were posttreatment. Patients on methimazole therapy received 20-30 mg/day for the first phase, and the dose was reduced to 5-15 mg when patients achieved remission. Patients treated with propylthiouracil took 300-500 mg/day for the first phase and 25-100 mg for maintaining remission. Normal controls were free of autoimmune diseases, tumors, allergies, infectious diseases, and acute or chronic visceral diseases. The clinical information is indicated in Table 1. For lncRNA sequencing, five additional female GD patients and five matched female controls were randomly enrolled owing to the higher incidence of GD in females [4]. The clinical features of volunteers for sequencing are shown in Supplemental Table S1. The Ethics Committee of the Affiliated People's Hospital of Jiangsu University (No. K-20200012-Y) authorized this study. All participants provided written informed consent.

### 2.2. Laboratory Measurements

Serum samples were collected for the detection of thyroid function indicators. The levels of free triiodothyronine (FT3), free thyroxine (FT4), thyrotropin (TSH), thyroglobulin antibody (TgAb), and thyroperoxidase antibody (TPOAb) were measured by an LDX-800 system (Beckman Coulter, CA, USA). The levels of TRAb were measured by a Cobas 6000 system (Roche, Basel, Switzerland) based on the manufacturer's protocol.

### 2.3. Cell Isolation

Fresh human peripheral blood was collected by using an EDTA-K^2^ anticoagulant tube (Becton Dickinson, Sparks, USA). Then, the PBMCs of all subjects were separated by lymphocyte separation medium (Tianjin Haoyang Biological Technology Co., Tianjin, China) within 4 hours according to the manufacturer's instructions.

### 2.4. Identification of Differentially Expressed Genes

The PBMCs of five GD patients and five normal controls were lysed in 500 *μ*l of lysis buffer. Then, total RNA was extracted by using an RNA-Quick Purification Kit (YiShan Biotech, Shanghai, China) following the manufacturer's instructions. The RNA concentration was measured on NanoDrop ND-1000 instrument (Thermo Fisher Scientific, Waltham, MA, USA), and RNA integrity was measured by denatured agarose gel electrophoresis. These RNA samples were subsequently subjected to high-throughput sequencing of lncRNAs on the Illumina HiSeq platform following standard procedures (Cloud-Seq Biotech Ltd. Co., Shanghai, China). High-quality reads were obtained from raw sequencing reads using Cutadapt software and were aligned to the human reference genome (UCSC HG19) using HISAT2 software. lncRNA expression profiles were analysed using fragments per kilobase of exon per million fragments mapped (FPKM) values [17]. The differentially expressed lncRNAs between the GD and NC groups were screened by fold change (FC) and *p* values. Gene Ontology (GO) (http://www.geneontology.org) enrichment and Kyoto Encyclopedia of Genes and Genomes (KEGG) pathway (https://www.genome.jp/kegg/) analyses were performed to predict the potential roles.

### 2.5. qRT-PCR

cDNA reverse transcription and quantitative real-time PCR (qRT–PCR) were performed as previously described [18]. The primers are summarized in Supplemental Table S2. The transcript levels of lncRNAs and mRNAs were normalized to *β*-actin.

### 2.6. ROC Curve Analysis

Receiver operating characteristic (ROC) curves were drawn using Prism 8 software (GraphPad Software, Inc., San Diego, USA). The *Y*-axis represents the true positive rate, indicated by sensitivity. The *X*-axis represents the false positive rate, indicated by 100%-specificity%. The area under the ROC curve (AUC), sensitivity, and specificity were used to evaluate the diagnostic efficacy of the curve.

### 2.7. Statistical Analysis

Statistical analyses were conducted using Prism 8, which was also used to generate the plots. Statistical analysis for two variables was performed using an unpaired Student's *t*-test when variables passed the normal distribution test; otherwise, the Mann–Whitney test was used. Correlations were performed using Pearson's correlation coefficient. A *p* value less than 0.05 was considered statistically significant (^∗^*p* < 0.05, ^∗∗^*p* < 0.01, and ^∗∗∗^*p* < 0.001).

## 3. Results

### 3.1. The Expression Profile of lncRNAs in GD

**Figure 1:**
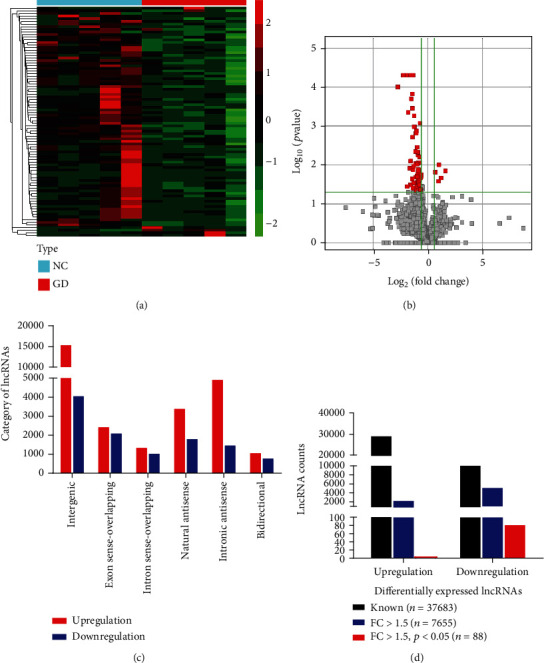
The expression profile of lncRNAs in GD. (a) Hierarchical clustering of differentially expressed lncRNAs in PBMCs between the GD and NC groups. The red squares represent upregulated lncRNAs, and the green squares reveal downregulated lncRNAs. (b) Volcano plot of dysregulated lncRNAs between the two groups. The red dots indicate significantly differentially expressed lncRNAs (FC > 1.5 and *p* < 0.05). (c) The category of dysregulated lncRNAs in GD. (d) A total of 27,354 upregulated and 10,329 downregulated lncRNAs were identified in the GD group compared with the NC group (black). Among them, 2,469 lncRNAs showed upregulated expression and 5,186 showed downregulated expression with over a 1.5-fold change in GD (blue). Eighty-eight significantly differentially expressed lncRNAs, including 5 upregulated and 83 downregulated lncRNAs, were identified (FC > 1.5 and *p* < 0.05) (red). Each data point represents an individual subject.

To investigate the role of lncRNAs in GD patients, we performed lncRNA sequencing in PBMCs to identify the dysregulated lncRNAs in GD (GEO ID: GSE197637). Figures 1(a) and 1(b) illustrated the hierarchical clustering and volcano plot of the differential expression of lncRNAs between the GD and NC groups. These lncRNAs were divided into six categories. Among the upregulated lncRNAs, intergenic lncRNAs accounted for 54.01%, exonic sense-overlapping lncRNAs accounted for 8.51%, intronic sense-overlapping lncRNAs accounted for 4.75%, natural antisense lncRNAs accounted for 11.83%, intronic antisense lncRNAs accounted for 17.09%, and bidirectional lncRNAs accounted for 3.80%, whereas these kinds of lncRNAs accounted for 35.72%, 18.59%, 9.33%, 16.13%, 13.09%, and 7.15% in the downregulated lncRNAs, respectively (Figure 1(c)). A total of 37,683 lncRNA transcripts were identified in GD, including 27,354 upregulated lncRNAs and 10,329 downregulated lncRNAs. Among them, the FC of 7,655 lncRNAs was more than 1.5 in GD compared with NC, while 88 lncRNAs showed FC > 1.5 and *p* < 0.05 (Figure 1(d)). These data indicated that differentially expressed lncRNAs were present in PBMCs from GD patients.

### 3.2. GO Analysis and Pathways of Differentially Expressed lncRNAs

**Figure 2:**
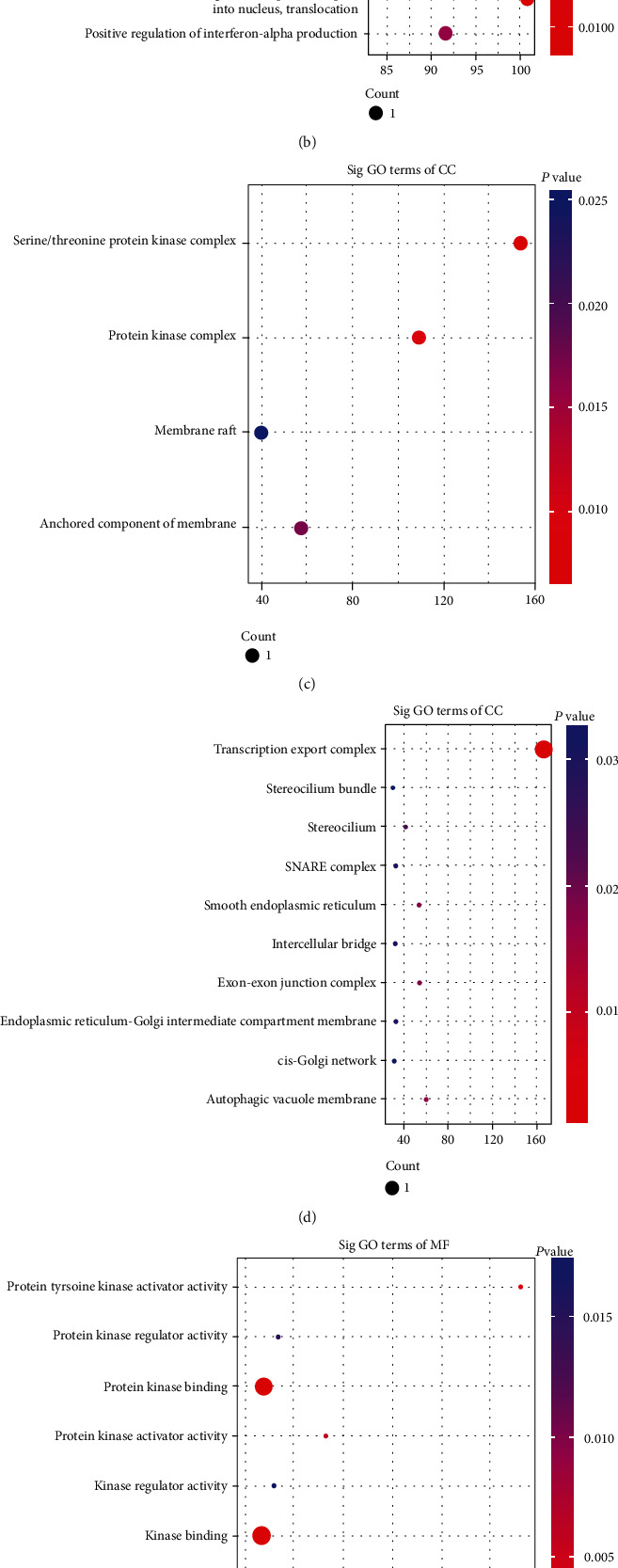
The top predicted functional terms of source genes regulated by differentially expressed lncRNAs in GD were obtained with GO analysis. They were categorized based on biological process (BP), cellular component (CC), and molecular function (MF). (a) The top GO terms of upregulated lncRNAs in BP. (b) The top GO terms of downregulated lncRNAs in BP. (c) The top GO terms of upregulated lncRNAs in CC. (d) The top GO terms of downregulated lncRNAs in CC. (e) The top GO terms of upregulated lncRNAs in MF. (f) The top GO terms of downregulated lncRNAs in MF.

**Figure 3:**
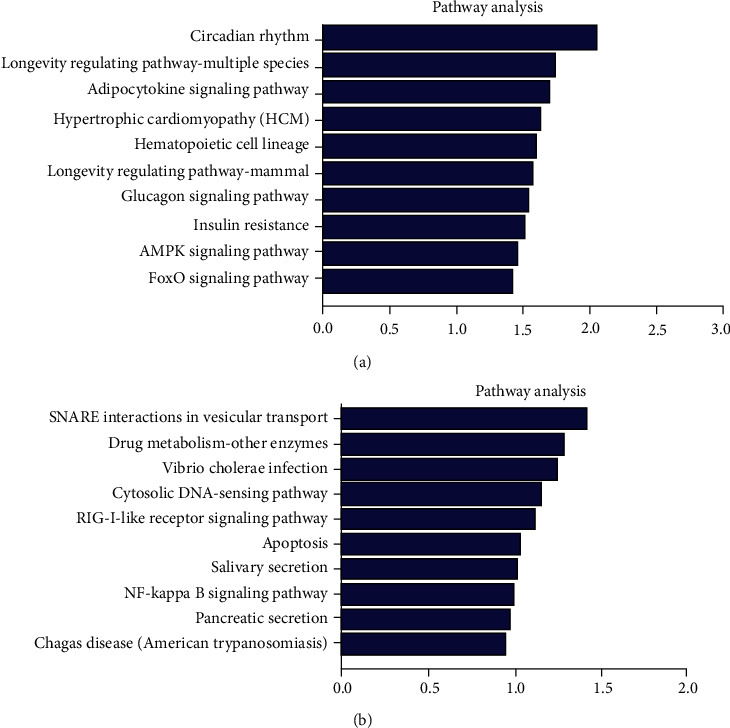
KEGG pathway analysis of differentially expressed lncRNAs. KEGG analysis showed 32 signalling pathways related to the dysregulated lncRNAs in GD. (a) The top 10 KEGG pathways of overexpressed lncRNAs. (b) The top 10 KEGG pathways of downregulated lncRNAs.

GO and KEGG pathway enrichment analyses were performed to predict the biological functions of dysregulated lncRNAs in GD. The GO analysis was divided into three categories: biological process (BP), cellular component (CC), and molecular function (MF). We observed that 148 GO terms of upregulated lncRNAs were statistically significant. The top GO enrichment terms are shown in Figure 2. These upregulated lncRNAs were mainly enriched in the regulation of nephron tubule epithelial cell differentiation (BP) (Figure 2(a)), serine/threonine protein kinase complex (CC) (Figure 2(c)), and protein kinase binding (MF) (Figure 2(e)). Meanwhile, GO analysis also indicated that 246 GO terms of downregulated lncRNAs were statistically significant, and the most prominent GO terms of downregulated lncRNAs were Golgi organization in BP (Figure 2(b)), transcription export complex in CC (Figure 2(d)), and SUMO binding in MF (Figure 2(f)). KEGG pathway analysis revealed that 13 pathways were associated with increased lncRNAs and 19 pathways were related to decreased lncRNAs in GD. These enriched pathways included the AMPK signalling pathway, FoxO signalling pathway, NF-kappa B signalling pathway, RIG-I-like receptor signalling pathway, cytosolic DNA-sensing pathway, and TNF signalling pathway. The top 10 KEGG pathways of dysregulated lncRNAs are shown in Figure 3.

### 3.3. Validation of the Selected lncRNAs

**Figure 4:**
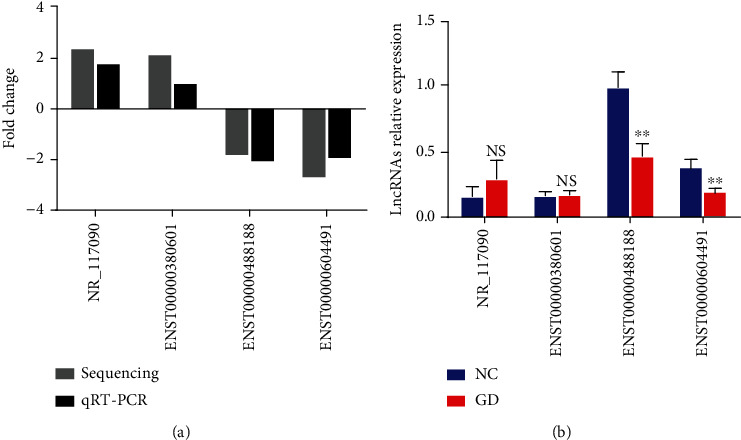
Validation of the selected lncRNAs. PBMCs were obtained from 26 GD patients and 27 normal controls. (a) The fold change of four selected lncRNAs between sequencing data and verified results by qRT-PCR. (b) qRT-PCR analysis of the expression of NR_117090, ENST00000380601, ENST00000488188, and ENST00000604491. ^∗∗^*p* < 0.01.

To further investigate the idiographic role of lncRNAs, we selected two upregulated lncRNAs (NR_117090 and ENST00000380601) and two downregulated lncRNAs (ENST00000488188 and ENST00000604491) for the validation by expanding the sample size based on the ranking order of multiple integrated factors, including the potential association with GD, the fold change, the FPKM, and the uniform expression between samples. Our data revealed that the expression trend of four lncRNAs was consistent with the sequencing results (Figure 4(a)), but only ENST00000488188 and ENST00000604491 levels were significantly attenuated in the PBMCs of GD compared with NC (Figure 4(b)).

### 3.4. ROC Curve Analysis of lncRNAs

**Figure 5:**
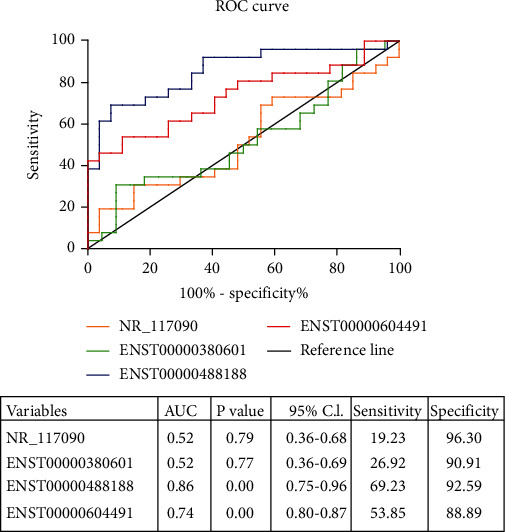
ROC curve analysis was performed to assess the diagnostic value of verified lncRNAs (NR_117090, ENST00000380601, ENST00000488188, and ENST00000604491). The evaluation indicators included AUC, *p* value, 95% C.I., sensitivity, and specificity.

ROC curve analysis was performed to assess the diagnostic value of selected lncRNAs. Our data indicated that ENST00000488188 and ENST00000604491 could distinguish the GD group from the NC group. The AUC of ENST00000488188 was up to 0.86 (*p* < 0.001), which was more than those of ENST00000604491 (AUC = 0.74; *p* < 0.01), NR_117090 (AUC = 0.52; *p* = 0.79), and ENST00000380601 (AUC = 0.52; *p* = 0.77). ENST00000488188 showed higher sensitivity (69.23%) and specificity (92.59%) than ENST00000604491 (sensitivity (53.85%) and specificity (88.89%)) (Figure 5). These data demonstrated that ENST00000488188 and ENST00000604491 could potentially differentiate patients with GD from healthy subjects, and ENST00000488188 might be more valuable as a potential biomarker of GD.

### 3.5. Correlation between ENST00000604491 Expression and Clinical Indicators

**Figure 6:**
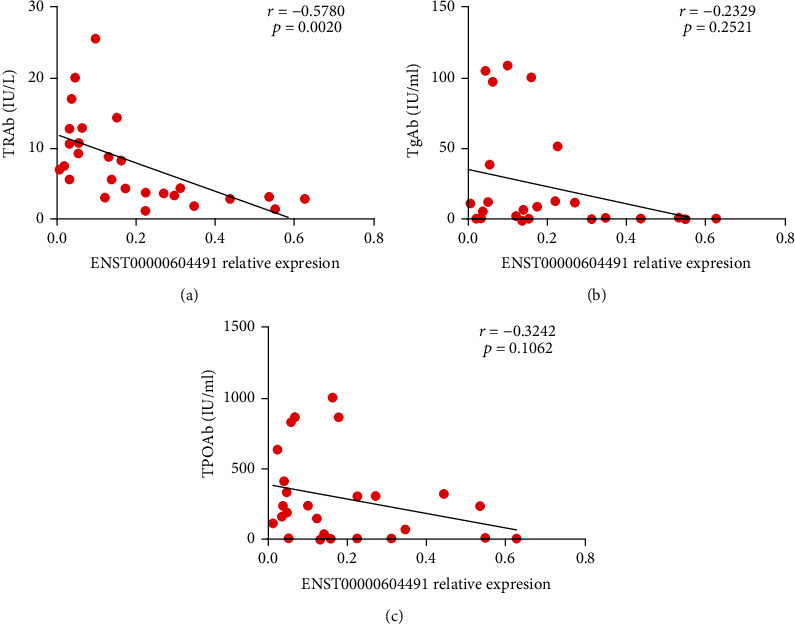
Correlation between ENST00000604491 expression and clinical indicators. The correlation between ENST00000604491 expression and serum levels of (a) TRAb, (b) TgAb, and (c) TPOAb in GD patients. Each data point represents an individual subject.

We next analysed the relationship between the transcript levels of ENST00000488188 and ENST00000604491 and thyroid autoantibodies, including TgAb, TPOAb, and TRAb. ENST00000604491 showed a tendency of inverse correlation with the serum levels of TRAb (*r* = −0.5780; *p* = 0.0020) (Figure 6(a)), but not with the serum concentrations of TgAb (*r* = −0.2329; *p* = 0.2521) (Figure 6(b)) and TPOAb (*r* = −0.3242; *p* = 0.1062) (Figure 6(c)). In addition, there was no correlation between the transcript levels of ENST00000488188 and the serum concentrations of thyroid autoantibodies (data not shown). These results suggested that downregulated ENST00000604491 was associated with GD.

### 3.6. Correlation between ENST00000604491 and FOXP1

**Figure 7:**
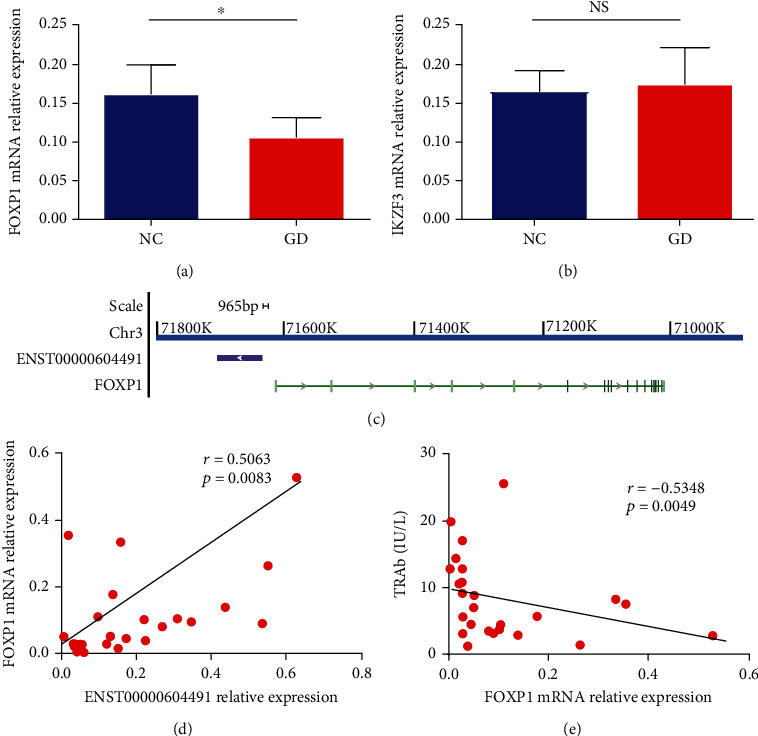
Correlation between ENST00000604491 and FOXP1. (a) The relative expression of FOXP1 mRNA in PBMCs between GD and NC. (b) qRT-PCR analysis of the transcript levels of IKZF3 in PBMCs from GD patients and controls. (c) Relative genomic positions of ENST00000604491 and FOXP1 on human chromosome 3. (d) The correlation between ENST00000604491 levels and FOXP1 levels in patients with GD. (e) The correlation between the transcript levels of ENST00000604491 and the serum levels of TRAb in GD. Each data point represents an individual subject. ^∗^*p* < 0.05.

The basic principle of cis target gene prediction is that the function of lncRNAs is related to the protein-coding genes adjacent to their location [19]. To address the function of ENST00000604491 and ENST00000488188, we found that FOXP1 was a potential regulatory gene of ENST00000604491, whereas ENST00000488188 potentially regulated IKZF3. We subsequently examined FOXP1 and IKZF3 expression in GD via qRT-PCR assay. Indeed, FOXP1 levels were significantly decreased in GD patients (Figure 7(a)). However, there was no change in IKZF3 expression between the GD and NC groups (Figure 7(b)). FOXP1 is a coding gene located at chromosome 3p13 and positioned 965 bp from the ENST00000604491 transcriptional start site (Figure 7(c)). To further assess the possible interaction between ENST00000604491 and FOXP1 in GD, we analysed the association between ENST00000604491 expression and FOXP1 levels and found that there was a notably positive correlation between the levels of ENST00000604491 and the levels of FOXP1 in GD patients (*r* = 0.5063; *p* = 0.0083) (Figure 7(d)). Meanwhile, an inverse correlation between FOXP1 levels and TRAb levels was shown in GD patients (*r* = −0.5348; *p* = 0.0049) (Figure 7(e)). These data suggested that ENST00000604491 was associated with FOXP1 expression in GD patients.

## 4. Discussion

GD is a multifactorial disease caused by the interaction of multiple environmental and genetic risk factors [20]. Numerous studies have demonstrated that abnormal immune responses of both T and B lymphocytes are essential for the development of GD [4]. However, unambiguous identification of the mechanisms of T cells and B cells dysregulation underlying GD has not yet been accomplished. lncRNAs have recently come into the spotlight with the publication of a number of studies in the past few years. Yin et al. performed a lncRNA microarray to identify AK021954, AB075506, and HMlincRNA1474 levels that were dysregulated in GD CD4^+^ T cells and might serve as novel biomarkers of GD [16]. Jiang et al. found that n335641, n337845, and TCONS_00022357-XLOC_010919 may participate in the proliferation and survival of B cells in GD [21]. Yao et al. focused on relapsed GD patients and found that three downregulated lncRNAs (NONHSAT093153.2, NONHSAT118924.2, and NONHSAT209004.1) were closely related to the recurrence of GD [15]. In the present study, we found a signature profile of numerous dysregulated lncRNAs (27,354 upregulated lncRNAs and 10,329 downregulated lncRNAs) in PBMCs from GD patients compared with normal controls by sequencing technology. Among them, intergenic lncRNAs accounted for the highest proportion. We subsequently investigated the potential functions of those differentially expressed lncRNAs via GO and KEGG enrichment analyses. GO analysis suggested that the upregulated lncRNA-associated GO term gene CD24 was expressed on regulatory B cells (Bregs) and could be responsible for GD development by breaking immune tolerance [22]. Downregulated lncRNAs were involved with a variety of GO term genes, such as FOXP1 and IKZF3, which are closely related to GD [23, 24]. Thirty-two KEGG pathways were identified to be associated with dysregulated lncRNAs in GD. Among these relevant pathways, the AMPK signalling pathway has been reported to be involved in GD progression [25]. In addition, it has been demonstrated that the FoxO signalling pathway includes multiple proinflammatory molecules and contributes to the formation and activation of regulatory T cells (Tregs), which have been proven to participate in the pathogenesis of GD [26, 27]. The RIG-I-like receptor signalling pathway has not been reported in GD, but the downstream Jak-STAT pathway regulates Th17 cells [28]. Additionally, the NF-*κ*B of the TNF signalling pathway and cytosolic DNA-sensing pathway have been shown to play an important role in GD by regulating the differentiation and functions of inflammatory T and B cells [29, 30]. These data screened the differentially expressed lncRNAs in GD and analysed the functions of these lncRNAs.

To further study the role of lncRNAs in GD, we selected NR_117090, ENST0000038060, ENST00000488188, and ENST00000604491 for validation. The transcript levels of ENST00000488188 and ENST00000604491 were remarkably decreased in GD. A previous study revealed that lncRNA RUX1-IT1 contributed to the differentiation of Th1 cells by regulating neural cell adhesion molecule (NrCAM) expression in GD [31]. A lncRNA named LPAL2 was found to modulate epidermal growth factor receptor (EGFR) signalling by targeting miR-1287-5p in orbital tissues from thyroid eye disease [32]. Gene expression is regulated by lncRNAs at multiple levels, and lncRNAs may broadly serve to fine-tune the expression of neighbouring genes [33]. To further investigate the functions of ENST00000604491 and ENST00000488188, we predicted FOXP1 and IKZF3 as the regulatory genes of ENST00000604491 and ENST00000488188, respectively. The present findings showed that FOXP1 expression was decreased in GD patients. However, the levels of IKZF3 did not change in GD patients compared with the normal controls. The relative position between a lncRNA and its adjacent genes is a key determinant of their regulatory relationship [11]. The ENST00000604491 transcript was positioned 965 bp downstream of the FOXP1 coding region. Consistently, ENST00000604491 expression was positively correlated with the transcript levels of FOXP1 in GD patients. FOXP1, belonging to the FOXP transcription factor family, is involved in the immune system by regulating T cells [34, 35]. FOXP1 can heterodimerize with FOXP3, which is necessary for the establishment and maintenance of Tregs and their suppressive function [36, 37]. In some well-studied cases, FOXP1 is essential for Treg cells by enforcing FOXP3-mediated regulation [38, 39], and the proportion of Treg cells is decreased in GD [40]. In addition, we found that FOXP1 expression was negatively correlated with the serum levels of TRAb in GD patients. Our results combined with previous studies suggest that ENST00000604491 may participate in the regulation of Treg cells in GD by regulating FOXP1.

Laboratory testing of thyroid autoantibodies is clinically used for the diagnosis and differential diagnosis of GD, which reflects disease status to some extent [41]. We analysed the relationship between ENST00000488188 and ENST00000604491 and thyroid autoantibodies. A notable observation in our study was the strong association of ENST00000604491 levels with serum TRAb levels in GD patients. Furthermore, ENST00000604491 showed the ability to distinguish GD patients from healthy controls. These data suggest that ENST00000604491 might be involved in the disease process of GD and serve as a potential biomarker for GD diagnosis. However, there are some limitations in the present study. First, the sample size for validation was too small. Second, we only preliminarily analysed the sequencing results and investigated the relationship between ENST00000604491 and FOXP1. Third, our data do not reflect ENST00000604491 levels in relapsed GD. Much work needs to confirm the conclusion with large cohorts of GD patients and *in vitro* and *in vivo* experiments.

## 5. Conclusions

In summary, our findings provide a research basis for the in-depth exploration of the function of dysregulated lncRNAs in GD. lncRNA ENST00000604491 was significantly downregulated, which might contribute to the reduction of FOXP1 in GD. The function of ENST00000604491 may provide new molecular mechanisms for the pathogenesis of GD and may serve as a potential biomarker of GD.

## Figures and Tables

**Table 1:** Clinical characteristics of GD patients and normal controls.

Variables	GD patients	Normal controls	Range
Number	26	27	—
Gender (M/F)	6/20	7/20	—
Age (year)	45 ± 13	45 ± 15	—
FT3 (pmol/liter)	8.74 ± 7.11	5.57 ± 1.57	3.28-6.47
FT4 (pmol/liter)	16.84 ± 12.52	10.79 ± 2.12	7.64-16.03
TSH (uIU/ml)	0.82 ± 1.47	2.25 ± 0.93	0.56-5.91
TgAb (IU/ml)	21.75 ± 36.67	0.15 ± 0.15	0-4
TPOAb (IU/ml)	274.95 ± 303.51	1.11 ± 1.58	0-9
TRAb (IU/liter)	8.03 ± 6.15	<0.80	0-1.75

Data correspond to the arithmetic mean ± SD. M: male; F: female; GD: Graves' disease.

## Data Availability

The sequencing datasets of lncRNAs can be found in GEO/GSE197637.

## Supplementary Materials

Supplementary Materials The clinical features of volunteers for lncRNA sequencing and the primers of qRT–PCR are shown in supplementary materials.
