# Supplementary material for: Comprehensive Analysis of lncRNA Expression Profile and the Potential Role of ENST00000604491 in Graves' Disease
Source: J Immunol Res. 2022 Apr 25;2022:8067464. doi: 10.1155/2022/8067464 (PMC9061081; doi:10.1155/2022/8067464)
Supplement: Supplementary Materials — The clinical features of volunteers for lncRNA sequencing and the primers of qRT–PCR are shown in supplementary materials. [file 8067464.f1.zip › 8067464-Table S1.docx]

**Table S1. Clinical features of GD patients and normal controls for lncRNA sequencing**

| Variables | Gender (M/F) | Age (year) | FT3 (pmol/liter) | FT4 (pmol/ liter) | TSH (uIU/ml) | TgAb (IU/ml) | TPOAb (IU/ml) | TRAb(IU/ liter) |
| --- | --- | --- | --- | --- | --- | --- | --- | --- |
| GD-1 | F | 31 | 23.40 | 69.14 | 0.02 | 218.80 | 976.00 | 2.31 |
| GD-2 | F | 32 | 10.17 | 34.46 | 0.01 | 19.70 | 81.70 | 4.77 |
| GD-3 | F | 62 | 10.10 | 44.56 | 0.01 | 295.10 | 161.70 | 5.20 |
| GD-4 | F | 53 | 14.05 | 27.29 | 0.22 | 8.90 | 37.90 | 16.68 |
| GD-5 | F | 50 | 11.32 | 38.44 | 0.01 | 36.20 | 353.30 | 6.32 |
| NC-1 | F | 30 | 5.53 | 12.63 | 1.46 | 0.10 | 0.50 | <0.80 |
| NC-2 | F | 27 | 4.23 | 11.37 | 2.18 | 0.30 | 0.40 | <0.80 |
| NC-3 | F | 47 | 4.01 | 10.77 | 1.93 | 0.20 | 0.90 | <0.80 |
| NC-4 | F | 50 | 5.11 | 9.31 | 2.42 | 0.10 | 0.60 | <0.80 |
| NC-5 | F | 49 | 5.24 | 10.47 | 2.03 | 0.10 | 0.50 | <0.80 |

Data correspond to the arithmetic mean ± SD. M: male; F: female. GD: Graves’ disease; NC: normal controls.
