# Supplementary material for: Comprehensive Analysis of lncRNA Expression Profile and the Potential Role of ENST00000604491 in Graves' Disease
Source: J Immunol Res. 2022 Apr 25;2022:8067464. doi: 10.1155/2022/8067464 (PMC9061081; doi:10.1155/2022/8067464)
Supplement: Supplementary Materials — The clinical features of volunteers for lncRNA sequencing and the primers of qRT–PCR are shown in supplementary materials. [file 8067464.f1.zip › 8067464-Table S2.docx]

**Table S2. Primer sequences of the genes in the study**

| Primer ID | Sequence (5’ - 3’) | | |
| --- | --- | --- | --- |
| NR_117090 | | Forward | GGTCGCACTGGAATTCGCAG |
|  |  | Reverse | GTTGTTTCACTGGAATAAATCCCC |
| ENST00000380601 | | Forward | AAGTTGCGGCTGATGAGGAA |
|  |  | Reverse | GAACTAATTCGAGGTTGGGTGTC |
| ENST00000488188 | | Forward | AAATGACTGATCCCACCGCC |
|  |  | Reverse | GTCTGCTTTGTAAATGACCTGCT |
| ENST00000604491 | | Forward | TGGACATTTACCACCTGACGA |
|  |  | Reverse | GATATGGCAAAAATCAGGACACC |
| FOXP1 | | Forward | CAATGGAGCATACCAACAGCAA |
|  |  | Reverse | GTGTACAGGATGCACGGCTT |
| IKZF3 | | Forward | GACCAAGCCATCAATAACGCC |
|  |  | Reverse | GGCTATGGGATACATGCTGCT |
| β-actin | | Forward | GAGTGTGGAGACCATCAAGGA |
|  |  | Reverse | TGTATTGCTTTGCGTTGGAC |
